# Effect of viscous dissipation and induced magnetic field on an unsteady mixed convective stagnation point flow of a nonhomogenous nanofluid

**DOI:** 10.1038/s41598-023-42593-1

**Published:** 2023-12-18

**Authors:** M. A. Aiyashi, S. M. Abo-Dahab, M. Daher Albalwi

**Affiliations:** 1 Department of Mathematics, College of Science, Jazan University, Jazan, P.O. Box. 114, 45142 Kingdom of Saudi Arabia; 2 Department of Mathematics, Faculty of Science, South Valley University, Qena, 83523 Egypt; 3 Yanbu Industrial College, The Royal Commission for Jubail and Yanbu, Jubail, 30436 Saudi Arabia

**Keywords:** Engineering, Materials science, Mathematics and computing, Physics

## Abstract

In the study, we investigate the numerical investigation of variable viscous dissipation and source of heat or sink in mixed convective stagnation point flow the unsteady non-homogeneous nanofluid under the induced magnetic parameter. Considering similarity conversions, the governing of fundamental boundary of layer non-linear PDEs are transformed to equations of the non-linear differential type that, under appropriate boundary conditions, are numerically solved, and the MATLAB function bvp4c is considered to solve the resulting system. The obtained results are calculated numerically for non-dimensional velocity, temperature, and volume fraction and displayed graphically. Further, numbers of Nusselt and Sherwood and local Skin of friction have been produced and displayed by graphs. A comparison with previous results obtained neglecting the new parameters has been made to show the impact of new external parametes on the phenomneon. The obtained findings agree with those introduced by others if the magnetic field and viscous dissipation are neglected. The results obtained have an important applications in diverse field as chemical engineering, agriculture, medical science, and industries.

## Introduction

In recent decades, more importance have been made to the topic of viscous dissipation effect on unsteady mixed convection, because of its utilitarian aspects in diverse fields as medical chemistry, medicine, engineering, industries,..., etc. The classical fluid models indicate to exploiting nanofluids in thermal systems is growing day by day. Nanofluids having ultrafine solid particles promise new working fluids for application in energy devices. Many studies have been conducted on thermophysical properties as well as heat and fluid flow characteristics of nanofluids in various systems to discover their advantages compared to conventional working fluids. The applied mathematics branch that produces small particles, such as dust to go from the hot surface towards the cold surface is called thermophoresis. These phenomena offer several particle uses to remove the small particles from gas streams, finding curved paths ß of the particles of exhaust gas by combusting devices, as well as exploring depositing on turbine blades as the particulate material. Nanofluid properties depend on base fluid properties, particle distribution, particle dimension and geometry, and interfacial impacts of fluid-particles. Nanofluid microstructure details are essential for estimating the efficacy of their characteristics. Nanofluids are produced by methods with either one or two steps. These methods require sophisticated equipment that leads to increasing nanofluid production costs. Therefore, the nanofluids high cost has negative impact of their applications. Authors adopt two approaches for estimating the characteristics of nanofluids if the comprehensive data are unavailable: determining the lower and the upper bounds on the characteristics in effect from partial statistical information or according to reasonable assumptions, estimating properties on the fluid microstructures. Nanofluids have proved highly successful in more uses. How nanofluids act is based on the dispersion and stability of nanoparticles within a system. Ultrasonication, pH control, and surfactant have been utilized in the proper dispersion of nanofluid, in which ultrasonication or a part of these processes plays a key role in the improved dispersion and stability. Nanofluid is a friendly environment which gives higher efficiency than the current fluids. Moreover, the colloidal mixture of particles nanosized in a fluid base enhances transferring heat suited features in real applications. Dharmalingam et al.^1^ presented a mathematical and experimental study about transferring heat through nanofluids, as well as the chemical and physical features, and analyzed the opportunities and challenges of nanofluids in further studies. Furthermore, they reviewed nanofluid, the latest research, its important heat transfer mechanisms, and development in nanofluids. Even though the nanofluid usage in the experimental application is critical due to forming sedimentation and clogging in the flow path, many of the mechanisms of transferring heat are discussed. Certainly, the use of liquids in nanofluids requires an inclusive understanding of their properties, such as thermal conductivity, specific heat, viscosity, etc. To expand nanofluid applications, we need further studies on the contributing factors for heat transfer and friction. The presence of suspended nanoparticles that have high thermal conductivity increases the higher thermal capacity of the liquid.

Several studies were onducted on the flow of stagnation-point because it has major applied uses in industry which makes the literature review in heat transfer enhancement by nanofluids, as well as studying the viscous fluid flows forced and free stagnation point^2–11^. The flow mixed convection phenomenon at the 2D geometric stagnation point for the mutilated physical characteristic has recieved much attention by several authors, such as^12–15^. Recently, Some new contributions have been discussed in Refs.^16–18^.

The flow-through boundary layer on the surface that moves continually of the 2D domain was at first modelled by Ref.^19^. The heat transfer and mass transfer convective process takes an important place because of the effects of buoyancy because of different concentration and temperature profiles, in turn. An important role has been played to address the phenomena transport, the thermal and mass diffusion taking place with the simultaneous buoyancy forces action. Mohamed et al.^20^ examined the finite element method technique (FEM) of a flow hydromagnetic and transferring heat of the fluid generation of heat over the embedded surface within the non-Darcian porousity considering chemical reactions. In Ref.^21^, the authors pointed out the chemical reaction and thermal radiations effect on transferring heat and mass in MHD flow of micropolar over a moving vertical porous plate within a porous medium that has a generation of heat feature. Effect of MHD and thermal radiation free convective flow of a polarized fluid through a porousity was discussed (see^22^). Analytical solution of the chemical reactions and thermal radiations impacts on MHD convection unsteady in a porous medium under heat source/sink were explored Abdel-Nasser et al.^23^. Mohamed et al.^24^ pointed out the unsteady magnetohydrodynamic convection boundary layer flow double-diffusive past a radiate vertical surface hot within a porous medium in considering chemical reactions and sink of heat. In Mohamed et al.^25^, the authors studied the chemical reactions, thermophoresis, variable viscosity, and heat source/sink impacts of on the non-Darcian mixed convective heat, as well as the flow of mass transfer. Carreau nanofluid radially stretching surface under effects of the magnetic field and the partial slip on an unsteady axisymmetric flow were discussed by Azama et al.^26^. Recently, the authors of Khashi’ie et al.^27^ highlighted the heat transfer and mixed convective flow of the dual stratified micropolar fluid produced using the permeable shrinking/ stretching sheet. Kumar and Srinivas^28^ discussed the Eyring Powell nanofluid flow unsteady hydromagnetic over stretching sheet inclined permeable under Joule heating and thermal radiation.

Nanofluid with magnetic field represents a colloidal suspension stable containing the base the solution, magnetic particles single-domain and surfactant. Because the motion of Brownian of the particles and the repulsion of the surfactant fosters the magnetic fluid and the resistance of magnetic particles to gravity sedimentation, is stable and depends on the size of magnetic nanoparticles and the physical properties of the based fluid (see Puga et al.^29^). Thus, thermal management and heat transfer have garnered widespread attention, because of their particular electronics devices, industrial applications, and thermal. The magnetic Fe3O4@CNT nanocomposites, the viscosity, thermal conductivity, and capacity of specific heat were determined by Shi et al.^30^. The adjustability, magnetic responsiveness, and recyclability of the transfer of heat demonstrated experimentally, and the magnetic nanofluid was prepared by the properties thermophysical of the nanofluid and hydrothermal method based on the temperature were explained^31^. The transpiration andiscous dissipation effect on the generation of entropy inflow of hybrid nan-ofluid over a radially non-linear stretching, the disk is investigated by Farooq et al.^32^. Ahmed et al.^33^ discussed the detail of the dual solution in a boundary layer flow of a fluid power-law over a moving plate permeable flat with viscous dissipation, heat generation/absorption, and thermal radiation. The combined electric field and magnetohydrodynamic impact on Maxwell’s nanofluid flow an unsteady over a stretching surface under considering the thermal radiation and heat effect variable explained by Khan^34^. The efficiency of heat exchange of magnetic nanoparticles suspension, magnetic nanofluid, in natural convection can be adjusted by changing the direction, form, and strength of magnetic field due to its properties of excellent magnetic (see^35,36^). However, the magnetic nano-particles thermal conductivity less than most nano-particles as CuO and Al2O3, limiting their wide applications in particularly in heat exchangers and the energy industry (see^37,38^). Hence, nano-composites of magnetic with a combination of magnetic property and higher thermal conductivity are an excellent choice for increasing the exchange of heat efficiency of natural convection more than other varies materials strongly. Reference^39^ discussed in detail the heat transfer convective magnetocontrollable of nanofluid via a straight tube.

Nanda et al.^40^ discussed the enhanced heat transmission in methanol-based AA7072/AA7075 tangent hyperbolic hybrid nanofluid flow along a nonlinear expandable surface. Kumar et al.^28^explained: A nanotechnology application considering the effect of electromagnetic induction on the heat transmission in engine oil-based hybrid nano and ferro-fluids. Sandeep et al.^41^ discussed the low and heat transfer in radiative MHD dusty-hybrid ferrofluids. Wang et al.^42^discussed the effects of nanoparticle aggregation and radiation on the flow of nanofluid between the gap of a disk and cone. Umavathi et al.^43^ investigated magnetohydrodynamic squeezing Casson nanofluid flow between parallel convectively heated disks. Kumar et al.^28^ studied heat transfer analysis in three-dimensional unsteady magnetic fluid flow of water-based ternary hybrid nanofluid conveying three various shaped nanoparticles: A comparative study. Oke et al.^44^ exploration of the effects of Coriolis force and thermal radiation on water-based hybrid nanofluid flow over an exponentially stretching plate. Gowda et al.^45^ pointed out three-dimensional coupled flow and heat transfer in non-newtonian magnetic nanofluid: An application of Cattaneo-Christov heat flux model. Kumar et al.^46^ discussed electromagnetic induction on the heat transmission in engine oilbased hybrid nano and ferrofluids: A nanotechnology application. Kumar et al.^47^ investigated the heat transfer analysis in three-dimensional unsteady magnetic fluid flow of water-based ternary hybrid nanofluid conveying three various shaped nanoparticles: A comparative study.

This study investigates the viscous dissipation impact of a mixed convection two-dimensional near-stagnation flow, and addition heat transfer with the magnetic parameter is considered through the normal direction of the plate. The authors’ knowledge was not considered by previous authors and never investigated in the scientific literature. The governing nonlinear PDEs boundary layer transformed to nonlinear differential governing equations considering the similarity transformations. The nonlinear differential equations under appropriate boundary conditions are numerically solved, and the MATLAB function bvp4c is considered to solve the resulting system. The obtained results are calculated numerically for non-dimensional velocity, temperature, and volume fraction and displayed graphically. Further, Nusselt and Sherwood numbers and local Skin friction are calculated and shown in the graphs. The results have been obtained are in agreement with the previous results obtained by others if the magnetic field and viscous dissipation are neglected.

## Mathematical analysis

This paper is a two-dimensional steady of coupled heat transfer by mixing the convective flow of incompressible viscous laminar fluid. Authors argue that the fluid is an unsteadily convective mixed stagnation point flow of nonhomogenous nanofluid and its various properties because of the restricted viscosity, density, and temperature. The variation density, and the buoyancy effect are considered in the equation of momentum (Boussineq’s approximation), and species of concentration far from the wall is $C_{\infty }$, is infinite similarly small and hence neglecting the Sort and Dufour numbers. Consider the *x*-axis in the plate direction, and the *y*-axis is normal on it. The external flow in the direction parallel to the plate inclined and has a uniform velocity $U_{\infty }$. The surface temperature becomes uniform $T_{w}$ that is more than the ambient temperature $T_{\infty }$. The species concentration at the surface is uniform at wall $C_{w}$, that is more than the ambient spices concentration $C_{\infty }$. The source/sink term of the chemical reaction is involved in the concentration equation.

**Figure 1:**
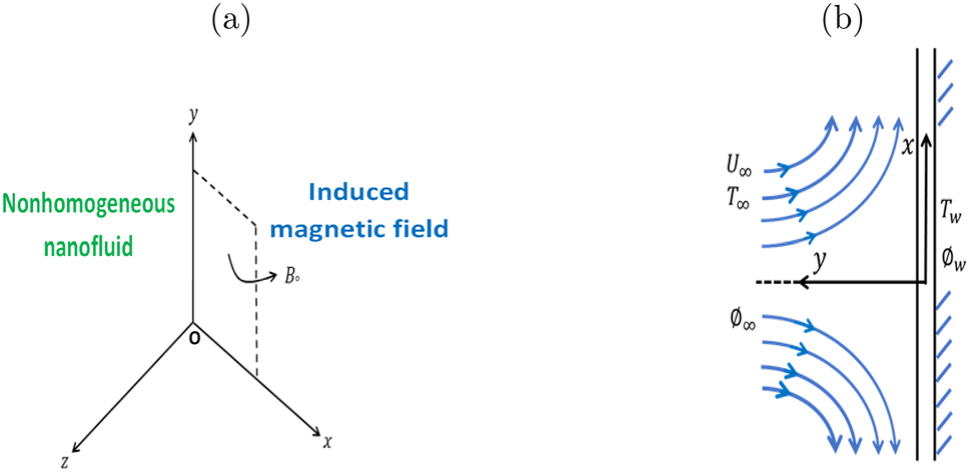
Geometrical shapes of (**a**) the magnetic field through fluid (**b**) flow configuration with coordinate system.

The derivation of magnetic term is introduced as follows:

The law of Ohm is written as (see^48,49^).

1 $$\begin{aligned} \textbf{j}+\dfrac{w_{e}\;\tau _{e}}{B_{\circ }}\;(\textbf{j}\times \textbf{B} )=\sigma \;(\textbf{v}\times \textbf{B}+\textbf{E}), \end{aligned}$$

where $\textbf{E}$ represents the intensity electric field, $\textbf{j}$ denotes the density of current, $\textbf{B}=(0,B_{\circ },0)$, $\textbf{E}= \textbf{0}$, $w_{e}\;\tau _{e}=m$ and $j_{y}=0$. Figure 1a shows the magnetic field in the coordinates system.

We have

2 $$\begin{aligned} \textbf{j}\times \textbf{B}&=B_{\circ }\;(-j_{z},0,j_{x}), \end{aligned}$$

3 $$\begin{aligned} \textbf{v}\times \textbf{B}&=B_{\circ }\;(-w,0,u). \end{aligned}$$

Substitute from Eqs. (2) and (3) into Eq. (1); yields** to**

4 $$\begin{aligned} (j_{x},0,j_{z})+m\;(-j_{z},0,j_{x})=\sigma \;B_{\circ }(-w,0,u). \end{aligned}$$

Equation (4) implies that

5 $$\begin{aligned} j_{x}&=\dfrac{\sigma \;B_{\circ }\;(mu-w)}{1+m^{2}}, \end{aligned}$$

6 $$\begin{aligned} j_{z}&=\dfrac{\sigma \;B_{\circ }\;(u+mw)}{1+m^{2}}. \end{aligned}$$

Hence, in the *x*-momentum equation, we add the force generated by the magnetic field, $\textbf{F}_{B}$

7 $$\begin{aligned} \textbf{F}_{B}=\textbf{j}\times \textbf{B}=(0-B_{\circ }\;j_{z},0,B\;j_{x}). \end{aligned}$$

From Eqs. (6) and (7) yields

8 $$\begin{aligned} F_{B_{x}}=-\dfrac{\sigma \;B_{\circ }^{2}}{1+m^{2}}\;(u+mw). \end{aligned}$$

$w=0$ since there is no flow in *z* direction,

9 $$\begin{aligned} F_{B_{x}}=-\dfrac{\sigma \;B_{\circ }^{2}}{1+m^{2}}\;u. \end{aligned}$$

Since $\tau _{e}<<$ and this is a result of a small magnetic field, the medium itself makes the electric conductivity small and $m=0$, therefore, Eq. (9) takes the form:

10 $$\begin{aligned} F_{B_{x}}=-\sigma \;B_{\circ }^{2}\;u. \end{aligned}$$

## Mathematical formulation

We take into account the unsteady 2D fused convective boundary layer flow of an electrically conducting viscous fluid near the recession point on a vertical plate. The fluid electrical conductivity is due to nanoparticles suspended in the fluid. This paper studies the transfer of heat and fluid flow near the wall, where point of stagnation occurs because of the flow divergence. Figure 1b shows configuring the problem.

The governing equations are

Continuity equation:

11 $$\begin{aligned} \frac{\partial u}{\partial x}+\frac{\partial v}{\partial y}=0. \end{aligned}$$

The equation of momentum:

12 $$\begin{aligned} \begin{aligned} \frac{\partial u}{\partial t}+u\frac{\partial u}{\partial x}+v\frac{\partial u}{\partial y}&=-\frac{1}{\rho _{f}}\frac{\partial p}{\partial x}+\nu _{f}\frac{\partial ^{2}u}{\partial y^{2}}-\frac{\sigma \beta ^{2}}{\rho _{f}}u \\ +\frac{1}{\rho _{f}}\bigg [&(1-\phi )\rho _{f\infty }g\beta _{T}(T-T_{\infty })-(\rho _{p}-\rho _{f_{\infty }})g(\phi -\phi _{\infty })\bigg ]. \end{aligned} \end{aligned}$$

Energy equation:

13 $$\begin{aligned} \begin{aligned} \frac{\partial T}{\partial t}+u\frac{\partial T}{\partial x}+v\frac{\partial T}{\partial y}&=\alpha \frac{\partial ^{2}T}{\partial y^{2}} \\ +\tau \bigg [D_{B}&\left( \frac{\partial \phi }{\partial y}\frac{\partial T }{\partial y}\right) +\frac{D_{T}}{T_{\infty }}\left( \frac{\partial T}{ \partial y}\right) ^{2}\bigg ]+\frac{\mu _{f}}{\rho _{f}c_{\rho }}\left( \frac{\partial u}{\partial y}\right) ^{2}, \end{aligned} \end{aligned}$$

Concentration equation:

14 $$\begin{aligned} \begin{aligned} {\frac{\partial \phi }{\partial t} + u\frac{\partial \phi }{\partial x} + v\frac{\partial \phi }{\partial y}}&={D_{B}\frac{\partial ^{2} \phi }{\partial y^{2}}} + {\frac{D_{T}}{T_{\infty }}\frac{\partial ^{2}T}{\partial y^{2}}}.\\ \end{aligned} \end{aligned}$$

In Eqs. (11)–(14), *u* and *v* denote the velocities of the component along the axis *x* and *y* direction, in order. $\rho _{f}$ denotes the base fluid of density, *T* represents temperature of the nanofluid. $\sigma$ and $\alpha$ represent the electrical of fluid and thermal conductivity, in order. $\beta$ denotes the coefficients of thermal expansion, the thermophortic diffusion coefficient denoted by $D_{T}$, $D_{B}$ suggests the coefficients of Brownian diffusiont. $\mu _{f}$ represents the viscosity of the nanofluid, $\rho _{p}$ describes the nanoparticles density. $\phi$ denotes the nanoparticle concentration and *g* denotes gravity-caused acceleration.

The generalized Bernulli‘s equation, in free stream, in Eq. (12) is

15 $$\begin{aligned} \frac{dU_{\infty }}{dt}+u_{\infty }\frac{dU_{\infty }}{dx}=-\frac{1}{\rho _{f}}\frac{dP}{dx}-\frac{\sigma \beta ^{2}}{\rho _{f}}U_{\infty }. \end{aligned}$$

Substituting Eq. (15) into Eq. ( 12) yields:

16 $$\begin{aligned} \begin{aligned} \frac{\partial u}{\partial t} + u\frac{\partial u}{\partial x} +v\frac{\partial u}{\partial y}&= \frac{dU_{\infty }}{dt} + u_{\infty }\frac{dU_{\infty }}{dx} + \frac{\sigma \beta ^{2}}{\rho _{f}}(U_{\infty }- u) + \nu _{f}\frac{\partial ^{2}u}{\partial y^{2}} \\ {}&\quad + \frac{1}{\rho _{f}} \bigg [(1-\phi _{\infty })\rho _{f\infty }g\beta _{T}(T-T_{\infty }) - (\rho _{f} - \rho _{f\infty })g(\phi - \phi _{\infty })\bigg ]. \end{aligned} \end{aligned}$$

Changing the nonlinear differential equations system into a set of ordinary differential equations system, the dimensionless variables become^50,51^,

17 $$\begin{aligned}&\eta =\bigg (\frac{a}{\nu _{f}(1-ct)}\bigg )^{\frac{1}{2}}y,\qquad \psi =\bigg (\frac{ a\nu _{f}}{(1-ct)}\bigg )^{\frac{1}{2}}xf(\eta ),&\nonumber \\&\phi =\frac{\phi -\phi _{\infty }}{\phi _{w}-\phi _{\infty }},\qquad \qquad \theta =\frac{T-T_{\infty }}{T_{w}-T_{\infty }}.&\end{aligned}$$

where the $\psi$ is the function of stream defined as $u=\frac{\partial \psi }{\partial y}$, $v=-\frac{\partial \psi }{\partial x}$. Thus, Eq. (11) in the model is fulfilled. The velocity components *u* and *v* have the following forms:

18 $$\begin{aligned} u= & {} \frac{a}{1-ct}xf^{\prime }, \end{aligned}$$

19 $$\begin{aligned} v= & {} -(\sqrt{\frac{a\nu _{f}}{1-ct}})f. \end{aligned}$$

### Boundary settings

The initial and boundary conditions are

20 $$\begin{aligned}{} & {} \ \ \text {at}\ \ t=0,\ \ u=v,\ \ T=T_{\infty },\ \ \phi =\phi _{\infty }, \nonumber \\{} & {} \ \ \text {at}\ \ x=0,\ \ t>0,\ \ T=T_{w},\ \phi =\phi _{w},\ \ u=u_{w}(x,t),\ \ v=V_{w}^{*}(T), \nonumber \\{} & {} \ \ \text {as}\ \ y\rightarrow \infty,\ \ t>0,\ \ u\rightarrow U_{\infty }(x,t),\ \ \phi \rightarrow \phi _{\infty },\ \ T\rightarrow T_{\infty }. \end{aligned}$$

The ambient fluid velocity far away from the considered wall

$$\begin{aligned} U_{\infty }(x,t)=\frac{ax}{1-ct}, \end{aligned}$$

and determines the stagnation point flow strength, $ct<1$ when $a>0$. Here, *a* and *c* are the constants that have dimension 1/*time*. The plate surface has stretching /shrinking along the velocity $u_{w}(x,t)=\frac{bx}{1-ct}$ where $b<0$ represents the shrinking of velocity and its stretching velocity when $b>0$ and *c* has dimension 1/*time*. The nano-fluid is non-homogeneous with average physical features of basic fluid and nanoparticles. At the plate $x=0$, the temperature *T* and the nanoparticle fraction $\phi$ fixed values $T_{w}$ and $\phi _{w}$, in order. The ambient values, attained as *y* becomes infinite of *T* and $\phi$ given by $T_{\infty }$ and $\phi _{\infty }$ in order.

- *The temperature of the wall takes*
  $$\begin{aligned} T_{w}(x,t)=T_{\infty }+\frac{T_{0}x}{(1-ct)^{2}}. \end{aligned}$$
  $T_{{\infty }}$ and $T_{0}$ denote the temperature of ambient fluid and the characteristic temperature, respectively.

- *Further, we assume that *
  $$\begin{aligned} V^{*}_{w} (t)=\bigg (\frac{\nu a}{1-ct}\bigg )^{\frac{1}{2}}V_{w}, \end{aligned}$$
  and $\phi _{w} (x,t)=\phi _{\infty } + \frac{\phi _{0} x}{(1-ct)^{2}}$ where the term $V^{*}_{w}$ denotes the uniform surface of mass flux. It is worth noting that $V^{*}_{w}> 0$ refers to injection wheres $V^{*}_{w}< 0$ means suction and it is an impermeable surface when $V^{*}_{w}=0$. A rigid vertical wall is located at $x=0$

## Numerical method

Employing the similarity transformation (17), Eqs. (13), (14) and (16) decline to these nonlinear ordinary differential equations:

21 $$\begin{aligned} f^{\prime \prime \prime }+ ff^{\prime \prime }- {f^{\prime }}^2 +1 +A (1 - f^{\prime }-\dfrac{1}{2}\eta f^{\prime \prime }) +M(1-f^{\prime })+\lambda (\theta - Nr\varphi )&=0, \qquad \end{aligned}$$

22 $$\begin{aligned} \theta ^{\prime \prime }+ Pr [ f\theta ^{\prime }- \theta f^{\prime }- A(2\theta + \dfrac{1}{2}\eta \theta ^{\prime })] + Nb\varphi ^{\prime }\theta ^{\prime }+ Nt{\theta ^{\prime }}^2 +{PrEcf^{\prime \prime }}^2&=0, \qquad \end{aligned}$$

23 $$\begin{aligned} \varphi ^{\prime \prime } + Scn [f\varphi ^{\prime } - \varphi f^{\prime } -A(2\varphi +\frac{1}{2}\eta \varphi ^{\prime })]-\frac{Nt}{Nb} \theta ^{\prime \prime }&=0. \qquad \end{aligned}$$

In this section, the MATLAB function bvp4c is applied to solve our model with boundary conditions.

The boundary settings become:

24 $$\begin{aligned}{} & {} \ \ \text {at} \ \ \eta =0, \ \ f^{\prime } =\frac{b}{a} =\varepsilon, \ \ f= -V_{w}, \ \ \theta =1, \ \ \varphi = 1, \nonumber \\{} & {} \ \ \text {at} \ \ \eta \rightarrow \infty, \ \ f^{\prime }\rightarrow 1, \ \ \theta \rightarrow 0, \ \ \varphi \rightarrow 0, \end{aligned}$$

in which the primes suggest differentiation concerning the independent variable $\eta$, $f^{\prime }$ denotes the velocity in dimensionless and $\theta$ tends the temperature in dimensionless . The physical parameters in this model are $A=\dfrac{c}{a}$ is an unsteadiness number, $\lambda =\dfrac{ G_{rx}\rho _{f_{\infty }}}{R_{ex}^{2}\rho _{f}}=\dfrac{(1-\phi _{\infty })g\beta _{f}(T_{w}-T\infty )x\rho _{f_{\infty }}}{(U_{\infty }(x,t))^{2}\rho _{f}}$ is bouyency or a mixed convection parameter, $M= \dfrac{\sigma B_{0}^{2}}{a\rho _{f}}$ is the magnetic parameter, $Nr=\dfrac{ (\rho _{p}-\rho _{f_{\infty }})(\phi _{w})-\phi _{\infty })}{(1-\phi _{\infty })\rho _{f_{\infty }}\beta _{T}(T_{w}-T_{\infty })}$ denotes the nanofluid ratio of buoyancy, $P_{r}=\dfrac{\mu _{f}}{\alpha _{f}}$ suggests the Pradentle number, $Sc=\dfrac{\nu _{f}}{D_{f}}$ represents the nanofluid Schmidt number. $Ec=\dfrac{U_{\infty }^{2}(x,t)}{C_{p}(T_{w}-T_{\infty })}$ represents the Eckert number, $Nb=\dfrac{D_{T}(T_{w}-T_{\infty })}{ D_{B}T_{\infty }(\phi _{w}-\phi _{\infty })}$ is the parameter Brownian of motion, $Nt=\dfrac{\tau D_{T}(T_{w}-T_{\infty })}{\alpha T_{\infty }}$ is the parameter of thermophoresis.

The utilized physical quantities denote the coefficient of the friction of skin $C_{f}$, Nusselt and the Sherwood numbers$Nu_{x}$ and $Sh_{x}$ that take the form as follows:

25 $$\begin{aligned} Cf_{x}=\frac{\nu _{f}}{\rho u_{w}^{2}}(\frac{\partial u}{\partial y} )_{y=0},\ \ Nu_{x}=\frac{xq_{w}}{k(T_{w}-T_{\infty })},\ \ Sh_{x}=\frac{ xq_{m}}{D_{B}(C_{w}-C_{\infty })}. \end{aligned}$$

In which the mass flux at surface $q_{m}$ and wall heat flux $q_{w}$ take the form

26 $$\begin{aligned} q_{m}=-D_{B}(\frac{\partial \phi }{\partial y})_{y=0},\ \ \ \ \ q_{w}=-k_{f}( \frac{\partial T}{\partial y})_{y=0}. \end{aligned}$$

Substitution Eqs. (17) and (18) into Eqs. (25) and (26), we obtain:

27 $$\begin{aligned} CfRe^{1/2}_{x} =f^{\prime \prime }(0),\ \ Nu_{x}Re^{-1/2}_{x} =-\theta ^{\prime }(0),\ \ Sh_{x}Re^{-1/2}_{x} =-\phi ^{\prime }(0),\qquad \qquad \end{aligned}$$

where $Re_{x}=\dfrac{U_{\infty }x}{\nu _{f}}$ suggests the Reynolds number.

## Results and discussion

**Figure 2:**
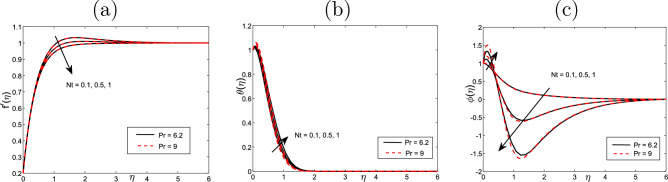
Impact of thermophoresis *Nt* for (**a**) the velocity $f^{\prime }$, (**b**) the temperature $\theta$, as well as (**c**) volume fraction $\phi$ profiles concerning the values $Scn=0.22$, $Nb=0.2$, $M=5$, $Ec=1$ and $A=Nr=Vw=\varepsilon =0.5$.

**Figure 3:**
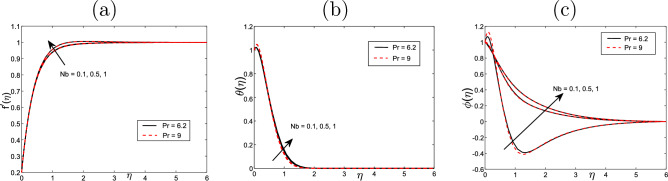
Effect of Brownian motion *Nb* (**a**) for the velocity $f^{\prime }$, (**b**) the temperature $\theta$ and (**c**) volume fraction $\phi$ profiles of the values $Scn=0.22$, $Nt=0.2$, $M=5$, $Ec=1$ and $A=Nr=Vw= \varepsilon =0.2$.

**Figure 4:**
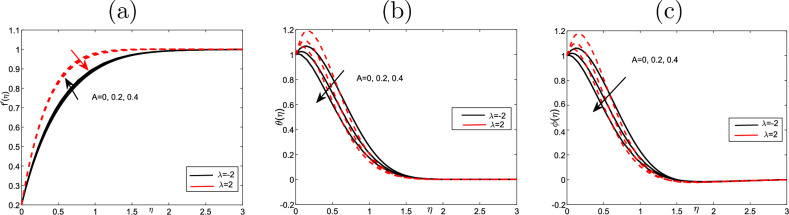
Effect of unsteadiness parameter *A* for (**a**) the velocity $f^{\prime }$, (**b**) the temperature $\theta$, as well as (**c**) volume fraction $\phi$ profiles of the values $Scn=0.22$, $Nt=Nb=\varepsilon =0.2$, $M=5$, $Ec=1$, $Pr=6.2$ and $Nr=Vw=0.2$.

**Figure 5:**
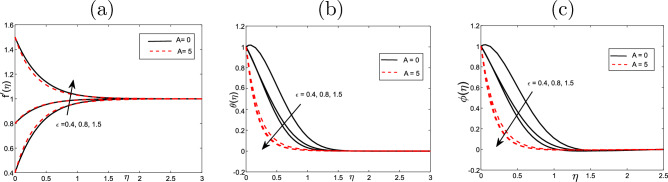
Effect of stretching/shrinking sheet parameter $\varepsilon$ for (**a**) the velocity $f^{\prime }$, (**b**) the temperature $\theta$ and (**c**) volume fraction $\phi$ profiles of the values $Scn=0.22$, $Nt=Nb=0.2$, $M=5$, $Ec=1$, $Pr=6.2$ and $\lambda =Nr=Vw=0.5$.

**Figure 6:**
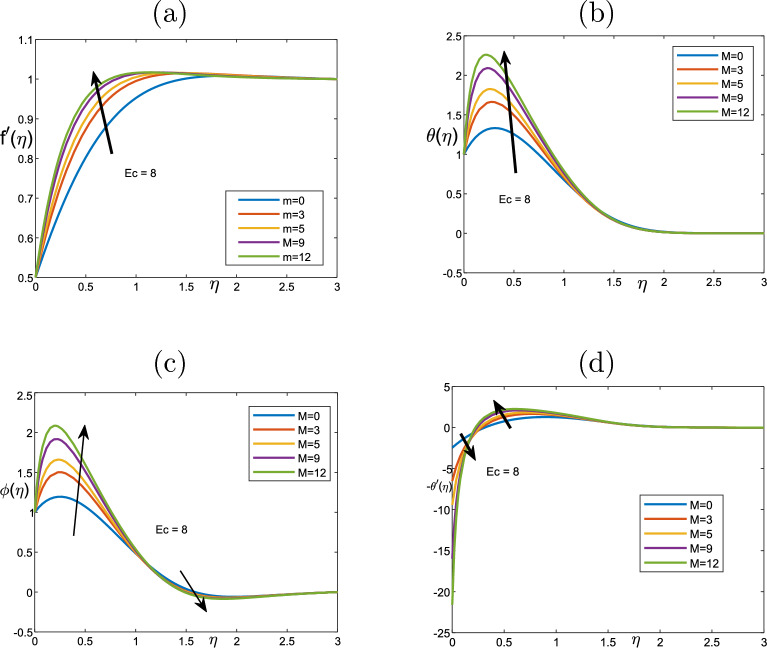
Effect of the variation of the magnetic parameter *M* for (**a**) the velocity $f^{\prime }$, (**b**) the temperature $\theta$, (**c**) volume fraction $\phi$, qs well as (**d**) the heat transfer profiles when $Scn=0.72$, $Nt=Nb=0.5$, $Pr=6.2$, $\lambda =1$, $Nr=0.65$, $A=0.3$ and $Vw=1$.

**Figure 7:**
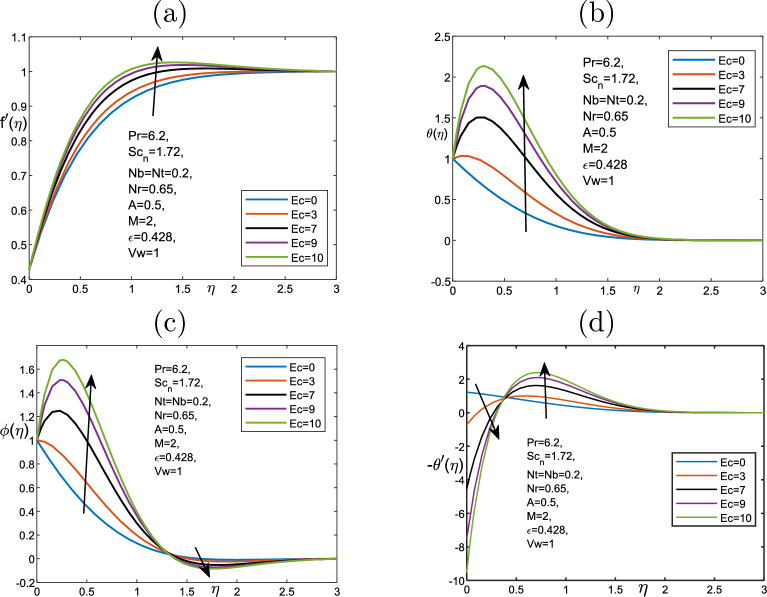
Impact of the variation of the Eckert number parameter *Ec* for (**a**) the velocity $f^{\prime }$, (**b**) the temperature $\theta$, (**c**) volume fraction $\phi$ and (**d**) the heat transfer $-\theta ^{\prime }$ for fixed parameters.

**Figure 8:**
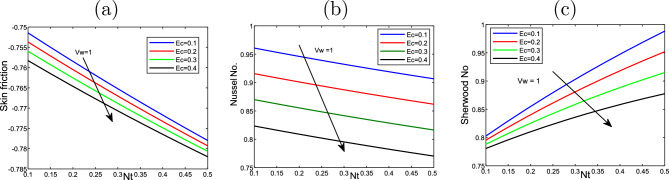
Impact of the variation of thermophoresis *Nt* and Eckert number *Ec* on (**a**) the Skin friction $f^{\prime \prime }(0)$, (**b**) Nusselt number $- \theta ^{\prime }(0)$, as well as (**c**) Sherwood number$-\phi ^{\prime }(0)$ at the value $Vw=1$.

**Figure 9:**
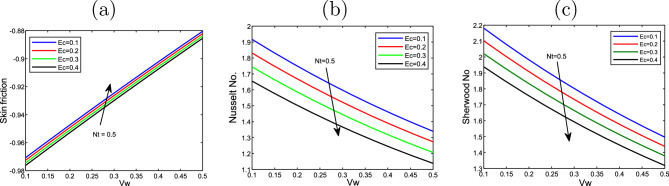
Effect of the variation of wall transpiration parameter *Vw* and Eckert number *Ec* on (**a**) the Skin friction $f^{\prime \prime }(0)$, (**b**) Nusselt number $-\theta ^{\prime }(0)$ along wih (**c**) Sherwood number$- \phi ^{\prime }(0)$ at the value of $Nt=0.5$.

**Figure 10:**
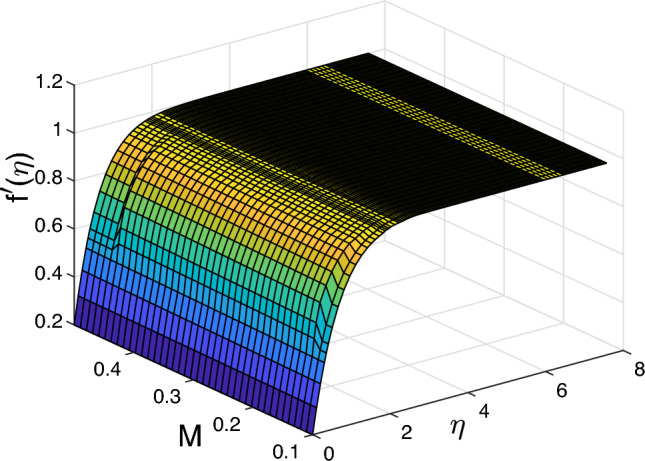
Various values of velocity $f^{\prime }$ for varying the magnetic *M* and $\eta$ in 3D.

**Figure 11:**
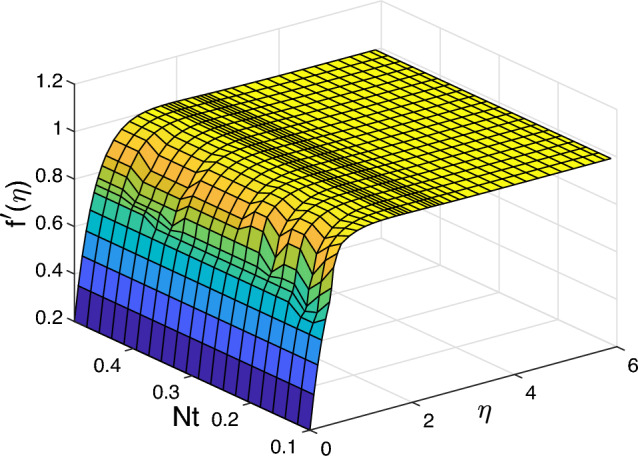
Various values of velocity $f^{\prime }$ for varying thermophoresis *Nt* and $\eta$ in 3D.

**Figure 12:**
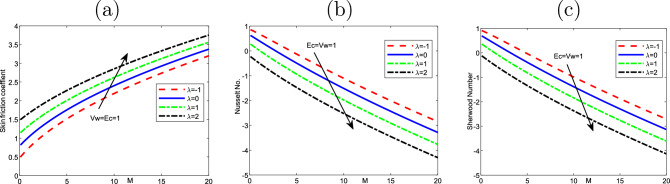
Effect of the variation of magnetic parameter *M* and buoyancy $\lambda$ on (**a**) the Skin friction $f^{\prime \prime }(0)$, (**b**) Nusselt number $-\theta ^{\prime }(0)$, as well as (**c**) Sherwood number $-\phi ^{\prime }(0)$ of the values of $Pr=6.2$, $Nt=Nb=Nr=0.1$,$A=0.5$, $\epsilon =0.2$ and $Scn=0.22$.

**Figure 13:**
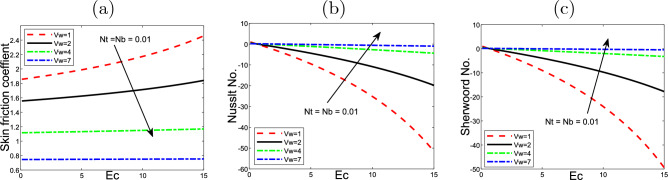
Effect of the variation of the parameter of wall transpiration *Vw* and Eckert number *Ec* on (**a**) the Skin friction $f^{\prime \prime }(0)$, (**b**) Nusselt number $-\theta ^{\prime }(0)$, as well as (**c**) Sherwoord number$-\phi ^{\prime }(0)$ of the values of $Pr=6.2$, $A=0.5$, $M=5$, $=Nr=0.1$, $\lambda =0.5$, $\varepsilon =0.2$ and $Scn=0.22$.

**Figure 14:**
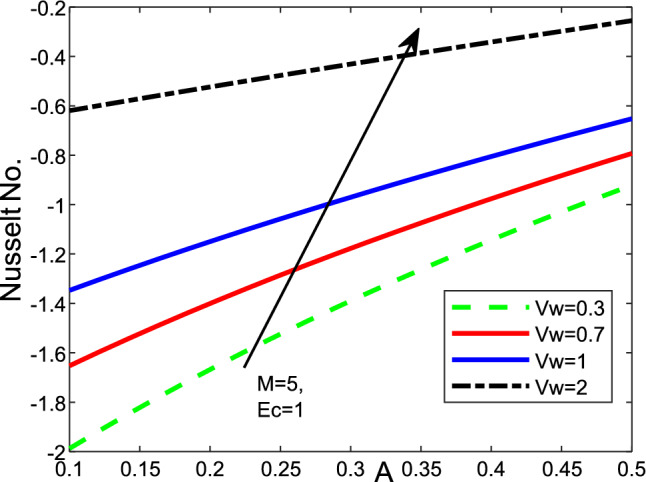
Effect of the unsteadiness parameter *A* on Nusselt Number for the values $Scn=0.22$, $Nt=Nb=\varepsilon =0.2$, $M=5$, $Ec=1$, $Pr=6.2$ and $Nr=0.2$.

**Figure 15:**
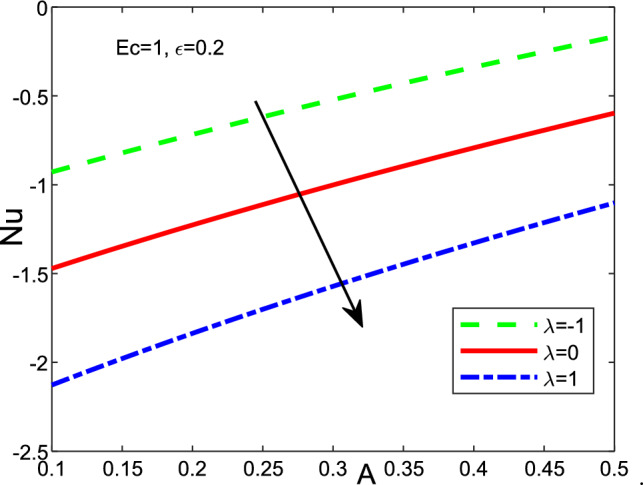
Effect of the parameter of mixed convection $\lambda$ on Nusselt Number when $Scn=0.22$, $Nt=Nb=0.2$, $M=5$, $Pr=6.2$ and $Nr=Vw=0.2$ for the variation of unsteadiness parameter *A*.

The numerical solutions of Eqs. (21)–(23) within the boundary settings (24) are displayed in Figs. 2, 3, 4, 5, 6, 7, 8, 9, 10, 11, 12, 13, 14 and 15. The findings of the coefficient of local Nusselt number and skin friction are comparable with those of Ramachandran^52^, Lok et al.^53^, and Kashani et al.^54^ for $\lambda =1,$ and various values of *Pr*, neglecting the parameters *Scn*, *Nb*, *Nt*, *Nr*, *A*, *M*, *Ec*, *Vw*,  and $\varepsilon$. We noticed that the comparison shows a perfect agreement, and our approach gives identical results with the results obtained by others, so we concluded that the current results obtained are appropriate. Tables 1 and 2 confirm the validity of the current results for the Nusselt number and coefficient of skin friction. Firstly, the velocity $f^{\prime }$ starts from its minimum value (i.e., $f^{\prime }=\frac{b}{a}=\varepsilon )$at the wall (i.e., $\eta =0)~$and increases approaches to the unity as $\eta \rightarrow \infty$  (far from the wall). The  volume fraction $\phi$ and profiles of temperature $\theta$ begin with the unity at the wall and decrease approaching zero as $\eta \rightarrow \infty .~$This physically indicates the increasing of velocity but vanishing temperature and volume fraction when the fluid is far from the wall. The result agrees with the experimental findings.

Figure 2 represents the thermophoresis impact *Nt* on the profiles of velocity $f'$, volume fraction $\phi ~$ and temperature $\theta$ for the values $Scn=0.22$, $Nb=0.2$, $M =5$, $Ec =1$ and $A=Nr=Vw=0.5$ A significant impact is noticed on the boundary layer regrading various values of Prandtl number $\Pr$ when it takes the values of 0.72 and 9. In this case the velocity $f'$ declines with the increased values of thermophoresis *Nt*, but the temperature $\theta$ and the volume fraction $\phi ~$ profiles rise. On the other hand, we show that the velocity declines with the increased values of *Pr* but volume fraction and the temperature decline. Similarly, Fig. 3 displays the impact of Brownian motion *Nb* for the velocity $f'$, the volume fraction $\phi ~$, and the temperature $\theta$ profiles. No significant impact on the temperature, velocity and volume fraction when the Pr takes the values 6.2 and 9 respectively. Only when the Pr takes the values 0.72 and 9 then the temperature and velocity increase with the increased values of *Pr* and *Nb*, but the volume fraction decreases. This indicates the the same impact of *Pr* and *Nb* on all physical quantities.

Figure 4 displays the impact of parameter of unsteadiness *A* on the velocity in *x* direction when $\lambda =- 2.2$. In this figure, we notice that the velocity is close to zero at the wall. When $\lambda =-2$ decreases, but when $\lambda =2$ increases. On the other hand, the temperature and the volume fraction profiles decline concerning the negative and positive values of $\lambda .~$ Physically, this means to the positive impact for the positive value of $\lambda$ but negative for the negative value of $\lambda$ and has applications in the related phenomena.

Figure 5 demonstrates the velocity profile behavior in the presence of $\varepsilon$ parameter (the stretching ratio of the sheet) and *A* (unsteadiness parameter) for two cases: one for a steady $A=0$ and other for $A=5$. The $\varepsilon <1,$ the velocity increases rapidly to reach the level of $\eta$. After that, it is identical with increase of $\eta$. In the same manner, when $\varepsilon >1,$ the velocity decreases dramatically and then becomes uniform with increase of $\eta$. Furthermore, the impact of $\varepsilon$ parameter and *A* on the temperature unsteadiness parameter can be shown in this figure. One can demonstrate that the volume fraction and the temperature decline with increasing $\varepsilon$ as well as the presence of parameter of unsteadiness *A*.

Figure 6 illustrates the parameter of magnetic *M* effect on the velocity, temperature and volume fraction profiles. The temperature only changes near the wall and then it stays uniform through the fluid as it increases. The thickness boundary layer is not under the impact of the magnetic field, due to the volume fraction profile. Therefore, the magnetic field affects the regions that have high concentration of the nanoparticles. The changes near the wall lead to the increment the transfer of heat, which can result in the change in the nanoparticles. Therefore, the temperature changes. Figure 7a plots that higher the parameter of the magnetic parameter is, the lower the velocity away from the wall becomes. Physically, we can conclude that when applying the magnetic field inside the boundary layer, it produces a type of resistance force called Lorentz body force that reflects the flow and slows the movement of the fluid. Figure 7 depicts the impact of Eckert number *Ec* on the temperature, the velocity, and the heat transfer. *Ec* statistically affects the fluid temperature because of improving *Ec* number, the improved viscous dissipation impact on the boundary layer of thermal. The *Ec* has a strong effect on velocity that we can deduce that the velocity along the surface increases and then becomes steady as $\eta$ increases. Furthermore, we can see that there is a small change in the boundary layer thickness on the transfer of heat near the wall and then the boundary layer declines to become uniform (no change).

Figure 8 plots the thermophoresis effect *Nt* and the Eckert number *Ec* on the friction of skin*Cf* $Nu_{x}$ also, the Sherwood number $Sh_{x}$. *Nt* and *Ec* rise, the skin friction along with Nusselt number decline. In contrast, the Sherwood number rises with rising *Nt* but declines with the large values of *Ec*.

Figure 9 displays the impact of Eckert number *Ec* and *Vw* on the friction of skin *Cf* $Nu_{x}$ and Sherwood number $Sh_{x}$.*Cf* $Nu_{x}$ rises when rises when *Ec* and *Vw* rise, while the Nusselt number and Sherwood number decline with the increasing of *Ec* and *Vw*. Figures 10 and 11 display 3D of parameter of magnetic *M* variation and $\eta,$ as well, variation of thermophoresis *Nt* and $\eta$ on the nondimensional velocity $f^{\prime }$ through the surface, respectively.

**Table 1:** The values of the skin friction coefficient concerning the different values of *Pr*, when $Scn=Nb=Nt=Nr=A=M=Ec=V_w=\varepsilon =0$ and $\lambda =1$.

$$CfRe^{1/2}_{x} =f^{\prime \prime }(0)$$				
*Pr*	Ramachandran et al.^52^	Lok et al.^53^	Kashani and Dinarvand^54^	Present
0.7	1.706376	1.7063	1.7063	1.7063
7	1.517952	1.5179	1.5177	1.5179
40	1.410094	1.4101	1.4103	1.4101

**Table 2:** Values of the local Nusselt number concerning various values of *Pr*, when $Scn=Nb=Nt=Nr=A=M=Ec=V_w=\varepsilon =0$ and $\lambda =1$.

$$Nu_{x}Re^{-1/2}_{x}=-\theta ^{\prime }(0)$$				
*Pr*	Ramachandran et al.^52^	Lok et al.^53^	Kashani and Dinarvand^54^	Present
0.7	0.76408	0.7641	0.7641	0.7641
7	1.72277	1.7224	1.7223	1.7224
40	3.10370	3.1011	3.1013	3.1011

It can be seen in the two cases that the magnetic *M* and thermophoresis *Nt* enhance the velocity near the wall. Then, it becomes a constant away through the surface as *M*, *Nt* and $\eta$ increase. Figure 12 is a plot for the various values of local Nusselt, friction of Skin, also, Sherwood number with magnetic *M* and mixed convection parameter regarding a fixed value of Ekert number *Ec* at 1. The friction of Skin rises with increasing the values of the parameter of mixed convection $\lambda$. On the other hand, there is a significant reduction in the Nusselt number and Sherwood number or heat transfer when the parameter of mixed convection rises with the parameter of magnetic field. Figure 13 displays the influence of wall transpiration parameter*Vw* and Eckert number *Ec* in the (a) Skin friction, (b) local Nusselt number, and (c) Sherwood number. Skin friction decreases with rising the values of *Vw* and increase *Ec*, but the local Nusselt number and Sherwood number rises when the values of *Vw* and *Ec* enhance. Furthermore, it is seen that Fig. 14 exhibits the effect of unsteadiness parameter *A* and parameter of wall transpiration *Vw* on the Nusselt number $-\theta ^{\prime }(0)$. Increasing *A* and*Vw* rises the local Nusselt number. Figure (15) reveals that the positive values of the parameter of fused convection $\lambda$ and parameter of unsteadiness *A* cause a reduction in the Nusselt number $-\theta ^{\prime }(0)$. *Nu* reduces with rising *A*,  but declines with increasing the fused convection parameter $\lambda .$

The obtained findings agree with those introduced by others if the magnetic field and viscous dissipation are neglected. The results obtained have important applications in diverse field as a chemical engineering, agriculture, medical science, and industries.

Finally, if the viscosity is neglected, the obtained results in this study reduce the results obtained by Hamad et al.^9^. Furthermore, if the magnetic field induced vanishes and considering the flow of hybrid nanofluid over the non-linear radially enlarging disk. Those obtained findings deduce those of Farook et al.^32^.

## Conclusion

We examined how the magnetic field and the viscous dissipation parameters affected the 2D unsteady mixed convective boundary layer flow. We found some vital point in the obtained results, such as:

- Temperature surface $\theta$ as well as volume fraction $\phi$ increase when thermophorsis *Nt* increases.
- The dissipation of viscous *Ec* strongly impacts temperature $\theta$ by increasing its profile.
- The parameter of magnetic*M* strongly impact on the temperature $\theta$ and $f\prime$ near the wall.
- Eckert number *Ec* and thermophorsis *Nt* reduce the skin of friction $C_{f}$ and Nusselt number *Nu*,  but raise the Sherwoord number *Shx*.
- The paper provides a system for achieving a superior performance of viscous dissipation variable and a heat source or sink on the unsteady mixed convective stagnation point flow of a non-homogeneous nanofluid under the induced magnetic field, and illustrates a system of improving the transfer of heat in a heat exchanger by controlling the magnetic field parameter distribution.
- The temperature, velocity, volume fraction, Sherwood number,as well as Nusselt number are affected strongly by the magnetic field and viscous dissipation in an unsteady mixed convective stagnation point flow of the nonhomogenous nanofluid that indicated the applicability in diverse fields, such as medical engineering, nanotechnology, and petroleum extracting.

Our results are generalized, and the agreement of the results obtained by others was highlighted^9,32^ with neglecting the new impacts.

## Data Availability

The data sets used and/or analysed during the current study available from the corresponding author on a reasonable request.
